# Differences in the use of Medicare Advantage transportation benefits by primary care payment model and beneficiary needs

**DOI:** 10.1093/haschl/qxag180

**Published:** 2026-07-15

**Authors:** Melanie Canterberry, Jeremiah Rastegar, Eric T Roberts, Robert E Burke, Emily Boudreau, Andrea DeVries

**Affiliations:** Humana Healthcare Research, Inc., Louisville, KY 40202, United States; Humana Healthcare Research, Inc., Louisville, KY 40202, United States; Perelman School of Medicine, University of Pennsylvania, Philadelphia, PA 19104, United States; Perelman School of Medicine, University of Pennsylvania, Philadelphia, PA 19104, United States; Humana Healthcare Research, Inc., Louisville, KY 40202, United States; Humana Healthcare Research, Inc., Louisville, KY 40202, United States

**Keywords:** Medicare Advantage, supplemental benefits, value-based care

## Introduction

Transportation challenges can restrict access to healthcare and are more prevalent among underserved patients, including those with low-income status or disability.^1^ To address this barrier to care, many Medicare Advantage (MA) plans offer nonemergency medical transportation (NEMT) as a supplemental benefit. While medical transportation facilitates fewer missed appointments and less delayed care, successful use of NEMT benefits among patients in need requires awareness of benefit availability and ability to coordinate the service.^1,2^ Identifying enablers of transportation benefit use is important to promote healthcare access among patients with transportation needs.

Primary care can play a vital role in facilitating NEMT use by identifying patients with transportation barriers and connecting them to benefits. We sought to evaluate to what extent payment models for primary care practices with different risk levels are associated with use of the transportation benefit for MA beneficiaries overall, and specifically for those patients with low-income status or a disability, as these have been linked with transportation needs.^3^ We hypothesized that increasing financial risk at the practice level would be associated with greater use of transportation benefits, given these practices may have stronger population health infrastructure and stronger financial incentives to ensure care continuity.

## Methods

For this retrospective, cross-sectional study, we identified individual MA beneficiaries continuously enrolled in a Humana plan offering a NEMT benefit from January 1 to December 31, 2022. Primary care practices (defined using taxpayer identification number [TIN]) were included if they had the same payment model during the entire study period. We used Humana's administrative data to identify beneficiary characteristics and attribution to a primary care provider (PCP). Low-income status was identified from Medicare–Medicaid dual-eligibility and/or Part D low-income subsidy enrollment, and disability was identified from beneficiaries’ original reason for Medicare entitlement. All beneficiaries either selected or were assigned a PCP through their MA plan based on visit and other claims data over the previous 24 months. We used contract data to identify the primary care practice's payment model—specifically, fee-for-service (FFS), shared savings with upside-only financial risk, and shared savings with upside and downside financial risk (2-sided risk).

The outcome variable was use of the NEMT benefit in 2022. We used encounter data to construct a binary measure coded as 1 when the beneficiary had at least one use. We conducted analyses among the full population and among the subgroup of beneficiaries with low-income status or disability. We first examined unadjusted rates of NEMT utilization by PCP payment model. We then constructed generalized linear regression models estimating the association between provider payment model and transportation use, using a binomial distribution with a logit link function and standard errors clustered by the practice level (using TIN). The models adjusted for beneficiary age, sex, race and ethnicity, geographic region, rural status, original reason for Medicare entitlement (full model only), low-income status (full model only), plan type, RxRisk-V comorbidity score, frequency of encounters with the PCP organization, and transportation benefit richness (annual number of rides available) (see Supplement for more details). We calculated the odds ratios (ORs) associated with having a provider with 2-sided or upside-only risk compared to FFS, as well as marginal means of transportation use per 1000 beneficiaries to aid with interpretation.

This study was reviewed by the Humana Healthcare Research Human Subject Protection Office and deemed not to be human subjects research. We followed the Strengthening the Reporting of Observational Studies in Epidemiology (STROBE) reporting guidelines.

## Results

Among the study population of 1 555 526 MA beneficiaries, the mean (SD) age was 70.0 (10.4), 876 385 (56.3%) were women, 347 359 (22.3%) were Black, and 1 038 713 (66.8%) were White. About half (49.7%) of beneficiaries had low-income status or a disability. The largest proportion of beneficiaries had PCPs in practices with 2-sided risk (45.3%), followed by upside-only risk (35.9%) and FFS (18.8%) (see Supplement for population characteristics). Overall, 4.2% of the study population and 6.8% of those with either low-income status or a disability used the NEMT benefit. In unadjusted comparisons, use of the transportation benefit was highest among beneficiaries with 2-sided risk providers, overall and among beneficiaries with low income or disability (Table 1).

**Table 1:** Use of the transportation benefit by presence of social risk factors and primary care provider payment arrangement.

	*N*	Transportation users per 1000^b^	Difference^c^	
Variable			Unadjusted	Adjusted
All beneficiaries	1 555 526	42.30		
FFS	292 847	38.38	Ref.	Ref.
Upside-only	557 852	38.03	−0.35	1.62
Two-sided	704 827	47.32	8.94	9.40
Beneficiaries with low-income status or disability^a^	773 650	68.21		
FFS	178 937	56.23	Ref.	Ref.
Upside-only	280 261	63.16	6.93	3.29
Two-sided	314 452	79.54	23.31	16.63

Abbreviation: FFS, fee-for-service.

^a^Beneficiaries with low income (low-income subsidy or dual-eligibility) or disability and/or end-stage renal disease as the original reason for Medicare entitlement.

^b^Unadjusted rates of beneficiaries having any transportation benefit use reported per 1000 beneficiaries.

^c^Difference in use per 1000 beneficiaries with providers in risk-based models compared to FFS. Adjusted marginal differences estimated from generalized linear regression models binomial distribution with a logit link function and standard errors clustered by the primary care provider organization, and including beneficiary age, sex, race/ethnicity, geographic region, rural status, original reason for entitlement (full model only), low-income status (full model only), plan type, frequency of encounters with the primary care provider group, RxRisk-V comorbidity score, and annual number of rides provided by the transportation benefit.

The adjusted analyses were consistent with the pattern in unadjusted comparisons: the likelihood of NEMT use was higher when the beneficiary's PCP had 2-sided risk (OR 1.38 [95% CI, 1.28-1.50]) or upside-only risk (OR 1.07 [95% CI, 1.01-1.13]) compared to FFS. Similarly, among beneficiaries with low-income status or disability, transportation use was higher when the provider had 2-sided risk (OR 1.38 [95% CI, 1.27-1.49]) or upside-only risk (OR 1.07 [95% CI, 1.02-1.14]) compared to FFS. Compared to FFS, 2-sided risk was associated with a marginal effect of 9.40 more beneficiaries overall and 16.63 more beneficiaries with low income or disability using transportation per 1000 beneficiaries. Transportation utilization rates are displayed in Table 1.

## Discussion

In this cross-sectional study of MA beneficiaries on a plan with a NEMT supplemental benefit, use of the NEMT benefit was generally low. However, MA beneficiaries overall, and particularly those with low-income status or disability, used NEMT at higher rates when they were attributed to primary care practices participating in advanced payment models. These value-based payment models hold practices accountable for the provision of high-quality care at low cost. While these findings are associational, they suggest that infrastructure enabling preventive care and condition management goals in value-based care may promote utilization of supplemental benefits among patients with access barriers (eg, screening patients for social needs and connection to resources through provision of care and benefit navigation services).^4^ The low utilization rates are not unexpected given findings of limited presence of transportation needs,^5^ though the variation by provider payment model suggests opportunity to expand reach.

This study has limitations. We lacked data to assess individual variation in transportation needs or the extent to which NEMT needs were met across practices. Selection biases, including existing practice-level capacity, may influence provider selection into a payment model or patients’ selection of their providers. Elucidating mechanisms facilitating benefit use and determining generalizability to other supplemental benefits requires further research.

## Supplementary Material

qxag180_Supplementary_Data
