# Supplementary material for: Differences in the use of Medicare Advantage transportation benefits by primary care payment model and beneficiary needs
Source: Health Aff Sch. 2026 Jul 15;4(7):qxag180. doi: 10.1093/haschl/qxag180 (PMC13420523; doi:10.1093/haschl/qxag180)
Supplement: qxag180_Supplementary_Data [file qxag180_supplementary_data.zip › Supplement.pdf]

Differences in use of Medicare Advantage transportation benefits by primary care payment model and beneficiary needs

Supplemental Online Content

eMethods.

Model Variables

We used Jan 1, 2022 as the index date. We used membership data to extract age, sex, race and ethnicity, original reason for Medicare entitlement, Medicare-Medicaid dual eligibility, Part D low-income subsidy (LIS), and residential state and zip code. Race and ethnicity was assessed according to the CMS beneficiary race and ethnicity codes and categorized as Black, White, under-represented (including Asian, Hispanic, North American Native, and other race or ethnicity), and unknown owing to inaccuracies in classification for non-White and non-Black beneficiaries. Beneficiaries with dual-eligibility or LIS for any month of the study period were classified as having a social risk factor. The original reason for entitlement was categorized as aged or disability. We included ESRD in the disability designation. We classified beneficiary residence using state according to US Census geographic region. We matched patient zip codes to Rural-Urban Commuting Area (RUCA) codes, and categorized areas with RUCA codes 1-3 as 'Non-rural' and RUCA codes 4 or greater as 'Rural'. We used enrollment files to identify each members plan type as HMO, PPO, Dual special needs plan and other.

We used claims to calculate the beneficiaries' total number of encounters with their attributed primary care provider group (using taxpayer identification number). We used pharmacy claims from Jan 1, 2022-Dec 31, 2022 to calculate the RxRisk-V comorbidity score.[1] A pharmacy-based measure of clinical risk was chosen because it may be less subject to differences that could result from diagnostic coding patterns. We used plan information to create a measure of transportation benefit generosity using the number of rides provided. We created tertiled groups based on the number of rides.

1. Pratt NL, Kerr M, Barratt JD, et al. The validity of the Rx-Risk comorbidity index using medicines mapped to the anatomical therapeutic chemical (ATC) classification system. *BMJ open*. 2018;8(4):e021122.

eTable1

Characteristics of Beneficiaries with a Transportation Supplemental Benefit

	**Overall N=1,555,526**	**Low-income status or presence of disability[a] n=773,650**
**Age, mean [SD]**	70.04 [10.43]	65.66 [11.62]
**Sex, n (%)**		
Female	876,385 (56.3)	448,454 (58.0)
Male	679,141 (43.7)	325,196 (42.0)
**Race,[b] n (%)**		
Black	347,359 (22.3)	226,704 (29.3)
White	1,038,713 (66.8)	460,351 (59.5)
Underrepresented	127,046 (8.2)	66,299 (8.6)
Unknown	42,408 (2.7)	20,296 (2.6)
**Geographic region, n (%)**		
Northeast	74,625 (4.8)	42,896 (5.5)
Midwest	289,008 (18.6)	135,997 (17.6)
South	918,096 (59.0)	488,074 (63.1)
West	273,797 (17.6)	106,703 (13.8)
**Population Density, n (%)**		
Rural	219,780 (14.1)	138,775 (17.9)
Non-rural	1,335,460 (85.9)	634,793 (82.1)
Unknown	286 (0.0)	82 (0.0)
**Medicare-Medicaid Dual Eligible, n (%)**	497,904 (32.0)	497,904 (64.4)
**Low Income Subsidy Eligible, n (%)**	564,478 (36.3)	564,478 (73.0)
**Original reason for Medicare entitlement, n (%)**		
Aged	1,040,963 (66.9)	259,088 (33.5)
Disability and/or ESRD	514,561 (33.1)	514,561 (66.5)
**Plan type**		
HMO	958,682 (61.6)	333,456 (43.1)
PPO	253,281 (16.3)	107,823 (13.9)

Dual Special Needs Plan	323,583 (20.8)	323,583 (41.8)
Other	19,980 (5.8)	8,788 (1.1)
**PCP payment model; n(%)**		
Two-sided risk	704,827 (45.3)	314,452 (40.6)
Upside only risk	557,852 (35.9)	280,261 (36.2)
FFS	292,847 (18.8)	178,937 (23.1)
**RxRisk Comorbidity Score, mean [SD]**	5.17 [3.33]	5.99 [3.47]
**Outpatient encounters with PCP Provider Group, Mean [SD]**	3.61 [3.66]	3.97 [4.08]
**Plan number of transportation rides (tertile)**		
<25	763,220 (49.1)	286,366 (37.0)
25-49	296,908 (19.1)	205,835 (26.6)
50+	495,398 (31.8)	281,449 (36.4)

SD, Standard deviation; ESRD, End-stage renal disease; HMO, Health management organization; PPO, Preferred provider organizations; PCP, Primary care provider.

[a]Beneficiaries with low-income (low-income subsidy or dual-eligibility) or disability and/or ESRD as the original reason for Medicare entitlement.

[b] Race and ethnicity were assessed according to the CMS beneficiary race and ethnicity codes and categorized as Black, White, Under-represented (including Asian, Hispanic, North American Native, and other race or ethnicity), Unknown.
